# The Effect of Bacterial Recombination on Adaptation on Fitness Landscapes with Limited Peak Accessibility

**DOI:** 10.1371/journal.pcbi.1002735

**Published:** 2012-10-25

**Authors:** Danesh Moradigaravand, Jan Engelstädter

**Affiliations:** 1 Institute of Biogeochemistry and Pollutant Dynamics, ETH Zurich, Zurich, Switzerland; 2 School of Biological Sciences, The University of Queensland, Brisbane, Queensland, Australia; University of Edinburgh, United Kingdom

## Abstract

There is ample empirical evidence revealing that fitness landscapes are often complex: the fitness effect of a newly arisen mutation can depend strongly on the allelic state at other loci. However, little is known about the effects of recombination on adaptation on such fitness landscapes. Here, we investigate how recombination influences the rate of adaptation on a special type of complex fitness landscapes. On these landscapes, the mutational trajectories from the least to the most fit genotype are interrupted by genotypes with low relative fitness. We study the dynamics of adapting populations on landscapes with different compositions and numbers of low fitness genotypes, with and without recombination. Our results of the deterministic model (assuming an infinite population size) show that recombination generally decelerates adaptation on these landscapes. However, in finite populations, this deceleration is outweighed by the accelerating Fisher-Muller effect under certain conditions. We conclude that recombination has complex effects on adaptation that are highly dependent on the particular fitness landscape, population size and recombination rate.

## Author Summary

The emergence and persistence of recombination is a long-standing open question in evolutionary biology. Most previous theoretical studies assumed relatively simple fitness landscapes, i.e., simple relationships between allelic states at different loci and fitness. By contrast, empirically determined bacterial and viral fitness landscapes reveal pervasive complex interactions between alleles at different loci. In this study, we explore the effect of recombination on adaptation on fitness landscapes where some trajectories leading to a global fitness peak are interrupted by genotypes of very low fitness. We find that in infinitely large populations, recombination generally reduces the rate of adaptation. However, in finite populations and under certain conditions, recombination can substantially speed up adaptation. Our study provides insights into the effect of recombination on more realistic fitness landscapes. Moreover, it helps gain a better understanding of the dynamics of the spread of adaptive genes in recombining bacterial populations during niche expansion and colonization of new habitats.

## Introduction

Sex and recombination are widespread phenomena in nature [1], [2]. The main effect of homologous recombination is to shuffle alleles at different loci. Therefore, for recombination to have an effect on the genetic composition of a population, a non-random association of alleles – called linkage disequilibrium (LD) – is required. Depending on how and what type of LD is generated in a population, recombination may accelerate or decelerate adaptive evolution of a population.

Among other factors, LD can be generated by epistasis and random genetic drift. Epistasis (in fitness) is a deviation of independent fitness effects of alleles at different loci. Magnitude epistasis refers to the case where the direction of selection is independent of the genetic background. Magnitude epistasis can either be positive (intermediate genotypes have a lower fitness than expected from the average of the extreme genotypes) or negative (higher fitness of intermediates). By contrast, with sign epistasis an allele can be selected for or against, depending on the allelic state at another locus [3]. Under adaptive evolution and in the absence of other LD generating forces, magnitude epistasis generates LD of the same sign [4]. Since negative LD implies a lower genetic variance for fitness and thus a reduced rate of adaptation, recombination can accelerate adaptation in this scenario by breaking up LD [5], [6]. However, empirical studies are ambivalent with respect to the prevailing form of epistasis in nature [7], [8], and some have reported strong positive epistasis [9]–[13].

In addition to epistasis, LD can also be generated through stochastic effects in finite populations [14]–[16]. In an asexual population, beneficial mutations arising in different individuals at different loci may compete against each other, which generates negative LD that impedes the adaptive process. This phenomenon is referred to as clonal interference and has been observed in bacterial and viral populations [17]–[20]. Recombination can bring the beneficial mutations arising in different genomes together, thereby increasing the efficiency of selection (the Fisher-Muller effect, [14], [15], [18], [21], [22]).

In most theoretical studies on the evolutionary consequences of recombination, either no epistasis or only a simple type of magnitude epistasis is considered under which deviations from independence of fitness effects are the same for all genotypes with the same number of deleterious mutations. The topology of these fitness landscapes is smooth. However, empirically determined fitness landscapes are often complex in that some landscapes exhibit pervasive sign epistasis [3], [23]–[28]. Here, only a limited number of mutational pathways to a fitness peak may be available [3], [25], [29], and local fitness peaks may be present [30], [31]. Recombination will in general have a strong impact on the rate of adaptation on such complex fitness landscapes [32]–[34], but it is largely unknown which types of fitness landscapes produce an accelerating or decelerating effect of recombination, especially when stochastic effects are taken into account. Most studies have considered only two loci, examining the effect of recombination in passing a fitness valley separating two peaks [3], [4], [35]–[37]. Recombination slows down adaptation in this case, or may even completely prevent the transition to the fittest genotype. To date, only a few studies have considered the effect of recombination on complex multilocus landscapes [32]–[34], [38], [39]. These studies show again that in general, recombination reduces the rate of adaptation [32]–[34], [but see 39].

In this study, we develop a mathematical/computational framework that allows us to examine the recombination effect on a special type of complex fitness landscapes that are characterized by sign epistasis. In these landscapes, we assume a single global fitness peak towards which the population can evolve, but we introduce a number of low fitness genotypes (‘LFG's) that make some mutational pathways inaccessible (or less accessible). Such limited peak accessibility has indeed been reported in some empirically obtained fitness landscapes (e.g., [25], [27]). Depending on the distribution of these genotypes, fitness landscapes adopt a variety of different topologies that may or may not involve local fitness peaks. Rather than focusing on obtaining analytical results for one or a few special landscape, we consider a broad range of different topologies and aim at obtaining a holistic view of the recombination effect on the adaptation on these fitness landscapes.

Motivated by the wealth of recent studies focusing on fitness landscapes and adaptation in bacteria [24], [27], [40]–[42], we assume a bacterial mode of recombination (through transformation); however, we expect our results to be applicable also to eukaryotic systems. We first show that in the absence of stochastic effects, recombination reduces the rate of adaptation in the vast majority of the fitness landscapes. However, in finite populations, recombination usually has an accelerating effect, indicating that advantages of recombination through stochastic effects may outweigh disadvantages that arise from epistasis.

## Methods

### Deterministic Model

**Figure 1:**
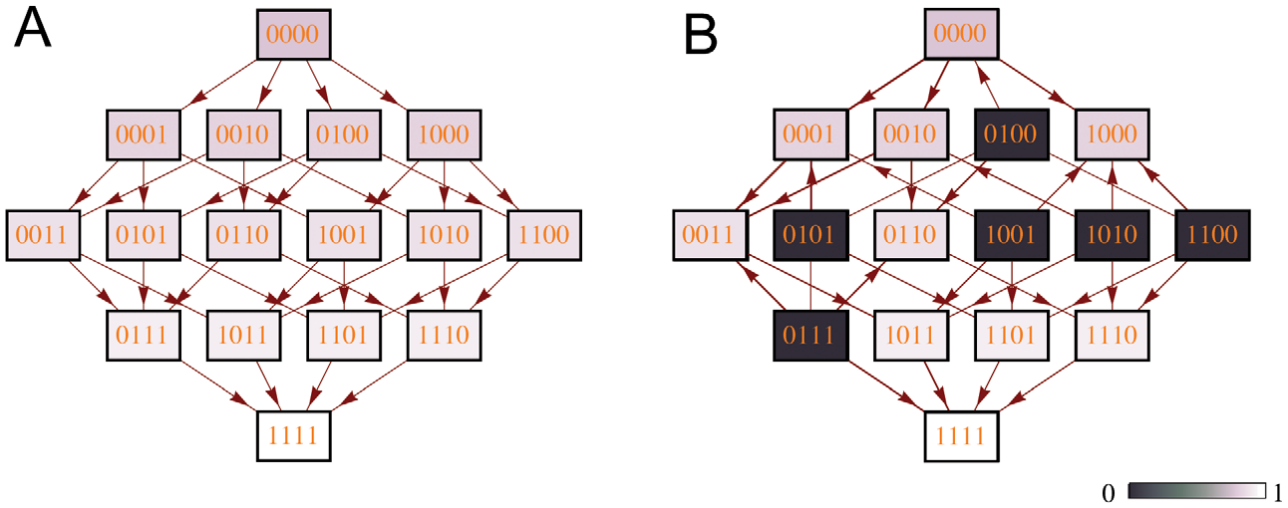
(A) A landscape with no LFG and (B) an example of a fitness landscape with 7 LFGs in the four-locus case. Darker colors correspond to lower relative finesses. Arrows show point mutation steps directed toward fitter genotypes.

We consider a continuous time model of a population of infinite size. Each individual is characterized by a genotype comprising *L* biallelic loci, with deleterious and beneficial alleles denoted by “0” and “1”, respectively. Hence, there are 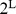 possible genotypes in the population, each of which is represented as a binary string of size 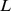 (Figure 1A). We denote the frequencies of these genotypes in the population by 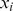, with 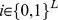. The population undergoes mutation, selection, and potentially recombination; these three processes will be specified below. We then compare the rate of adaptation in the presence and absence of recombination.

Mutations occur at a constant rate 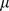 per locus. We assume that forward 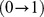 and backward 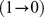 mutations occur at the same rate.

The fitness *m* of each genotype on the fitness landscapes in question is constructed in two steps. First, the basal landscape is a smooth, single-peaked landscape that potentially involves a one-dimensional type of magnitude epistasis. Specifically, we employ the following formula for determining fitness values:
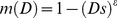
(1)Here, 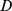 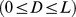 denotes the number of deleterious alleles (i.e., number of zeros) in the genotype. The fittest genotype, “1111”, is arbitrarily assigned a fitness value of 1, and all other genotypes have fitness values below one. 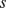 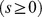 is the selection coefficient, here given by the reduction in fitness per deleterious allele. The strength of this epistasis can be adjusted by changing the value of the epistasis parameter, 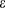. Values greater than one refer to the antagonistic action of beneficial mutations or negative epistasis and those less than one indicate synergistic mutations or positive epistasis. In a second step, low fitness genotypes (‘LFGs’) are introduced into the fitness landscape. These are defined as genotypes with relative fitness value of zero and may be introduced at different numbers and at different intermediate positions in the landscape (all genotypes except the least and the most fit, “0000” and “1111”). Note that because we are operating with Malthusian fitness values, a fitness value of zero of the LFGs does not imply that these genotypes are inviable, but rather it indicates their fitness is substantially lower than that of non-LFGs. We will refer to a specific configuration of LFGs as the fitness topography. Since both the parameters 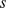 and 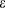 and the fitness topography determine a particular fitness landscape, there are infinitely many fitness landscapes for each fitness topography. An example for how these fitness landscapes are constructed is shown in Figure 1B.

Recombination is assumed to occur through transformation. Cells release free DNA into the environment, and we assume that that (1) all DNA fragments are of length 1 (a single allele), and (2) that the allele frequencies within the pool of free DNA are the same as in the bacterial population. These DNA fragments may be taken up by the bacteria at a rate 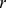 per locus and integrated into their genome at the homologous position. This way, an acceptor genome may be destroyed and replaced by a recombinant genome that incorporates a novel allele from a donor strain. This mode of recombination is different from that in eukaryotes in that it is always very localized, whereas a single crossover event in meiosis can break up linkage of a large number of genes on a chromosome. Nevertheless, the two modes of recombination are equivalent when only two loci are considered.

Integrating all of the above assumptions, we arrive at the following set of differential equations:
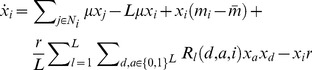
(2)The first two terms in these equations incorporate the mutational in- and out-flux of genotype frequencies into the model. Here, 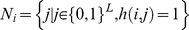 is the set of single-step neighbor genotypes of genotype 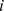, with 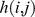 being the Hamming distance between sequences 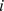 and 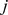. The third term gives the change in genotype frequencies as a result of fitness differences. Here, 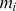
is the (Malthusian) fitness of genotype 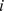, as determined by the fitness landscape, and 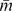 is the average fitness in the population. Finally, the last two terms arise through in- and out-flux of genotype frequencies through recombination. 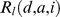 is an indicator function that takes the value 1 when recombination through single allele replacement at locus 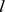 from donor genotype 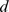 to acceptor genotype 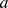 gives rise to the recombinant genotype 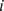; otherwise 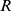 takes the value 0. This indicator function can be defined as
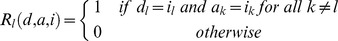
(3)We numerically solved the above differential equations (2) describing the model, using Mathematica 8 (Wolfram Research, Inc.). Here, we assumed that initially, the population consists only of the least fit genotype. Over time, beneficial alleles arise stepwise through mutation and may then rise in frequency through selection. The fixation time of the fittest genotype was defined as the time point at which the frequency of the fittest genotype exceeds 0.99. Since we generally assume low mutation and recombination rates, this threshold was always reached. The effect of recombination on the fixation rate of the fittest genotype was measured as the ratio, denoted by *T*_fix_, of the fixation time in the population with recombination to that in the population without recombination. Thus, *T*_fix_ is a measure for the effect of recombination on the rate of adaptation. In the Supplementary Online Material (Figure S9 in Text S1), we show fixation time of the fittest genotype also correlates strongly with the time it takes for the population mean fitness to increase to a certain threshold, indicating that our results are largely independent of which of the two measures for the rate of adaptation is used.

### Stochastic Simulation

In order to investigate the dynamics of adaptation in finite populations, we employed a modified version of a previously developed ‘hybrid algorithm’ [43]. Here, we model the different genotypes of the model as compartments of discrete sizes, and different events (birth, death, mutation, recombination) change the size of these compartments. The algorithm is based on the Moran model and incorporates Gillespie's exact algorithm [44], [45] for transitions in small sub-populations and coarse-grained 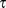-leaping [46] to simulate transitions in large sub-populations. This algorithm was shown to be accurate and computationally efficient for simulations of large population [43].

The size of compartment 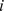 (i.e., the number of individuals with genotype 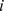 present in the population) is denoted by 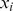. The following events and corresponding rate functions are used:

1. **Birth.** A type 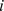 cell is born at rate 
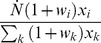
 where 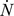 is a parameter that adjusts population growth and is here assumed to be constant, and 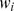 is the Fisherean fitness value of genotype 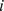. To convert fitness values from Malthusian to Fisherean, we used 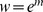, where 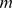 is the Malthusian fitness as defined in the previous section describing the deterministic model. Denoting 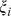 as the unit vector, the state change vector for this event is 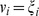.
2. **Death.** A type 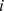 cell dies at rate 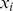 and the state change vector is 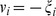.
3. **Mutation.** A type 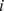 cell converts into a type 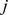 cell (with hamming distance of one) via point mutation with rate 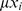, where 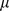 is the mutation rate. The state change vector for this event is 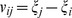.
4. **Recombination.** A type 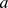 acceptor cell converts into a type 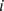 cell via recombination at locus 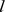 with rate 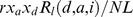
where 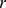 is the recombination rate, 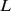 is the number of loci and 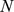 is the total population size. Here, 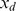 is the size of the subpopulation of donor type 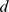 and 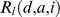 is an indicator function which takes value according to the equation 3. The state change vector is 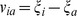.

The stochastic model converges to the deterministic one when the population size is very large and selection is moderate (see Figure S4 in Text S1). We again assumed that the population at the beginning consists of the least fit genotype only and simulate the process of adaptation until (near) fixation of the fittest genotype. To keep the simulation time low, the fixation time for the stochastic model was defined as the time at which the frequency of the fittest type exceeds 0.95 (but a 0.99 cut off did not change the final results). We performed 50 simulations in presence and absence of recombination for each landscape. We then calculated the means of fixation times in both cases. Thus, we obtained estimates for *T*_fix_, defined as the ratio of the mean fixation time in the population with recombination to that in the population without recombination.

## Results

We start by considering the deterministic evolutionary dynamics in our model, first for the simplest case of only two or three loci, and then for all four-locus fitness topographies. We then investigate the evolutionary dynamics for a subset of the four-locus fitness landscapes in the stochastic model.

### Deterministic Dynamics with Two and Three Loci

**Figure 2:**
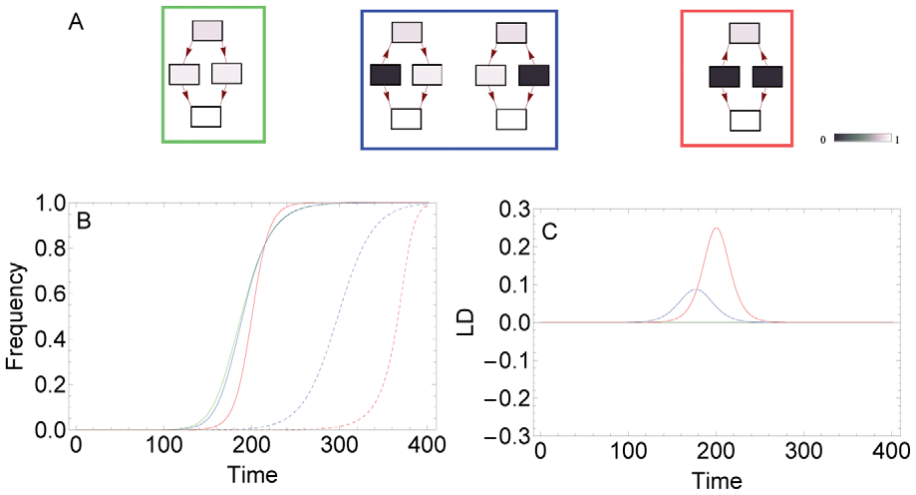
Dynamics in the two-locus model. Panel A shows four two-locus fitness landscapes with no LFG, one LFG (strong sign epistasis) and two LFGs (strong reciprocal sign epistasis). In B, the frequency of the fittest genotype is shown for the three types of fitness landscapes (green: no LFG, blue: one LFG, red: two LFGs), without recombination (solid lines) and with recombination (dashed lines). Plot C shows the corresponding LD dynamics of the three fitness landscapes without recombination. Parameters take the values 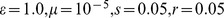.

In the two-locus case and for given parameters *s* and 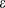, there are only three distinct fitness landscapes in our model depending on the number of LFGs (see Figure 2A): (1) the landscape with no LFG (which may be characterized by positive or negative magnitude epistasis, depending on the parameter 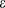), (2) two equivalent fitness landscapes with a single LFG (characterized by sign epistasis), and (3) the landscape with two LFGs (featuring reciprocal sign epistasis).

In the first landscape, it is well known that with negative epistasis, recombination accelerates fixation of the fittest genotype and with positive epistasis, recombination slows down the adaptive process [4], [5], [35]. When there is no epistasis, no LD builds up and hence, recombination has no effect on the dynamics (see Figure 2 for an example).

When there is a single LFG in the fitness landscape, this implies strong positive sign epistasis and therefore, we would expect that recombination decelerates fixation of the fittest genotype, which is in accord with simulations of this case (e.g., Figure 2B).

Finally, the case of two LFGs has been widely studied, for example in the context of compensatory mutations [4], [35], [47]. These previous studies have shown that recombination again slows down adaptation, as Figure 2B also reveals. Moreover, there is a critical recombination rate above which the fittest genotype does not spread at all, because it is broken down too rapidly by recombination into the two genotypes occupying the fitness valley. In order to derive this critical recombination rate, we neglected the mutational terms in equation (2) and performed a stability analysis of the fixed point corresponding to fixation of the 00 genotype (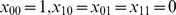). The eigenvalues of the Jacobian matrix evaluated at that fixed point are 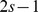 and 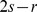. Assuming 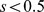 so that there is indeed a fitness valley, the critical recombination value above which both eigenvalues are negative and the fixed point is thus stable is therefore 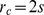. In other words, whenever there is a fitness valley of intermediate genotypes and the recombination rate is larger than the fitness difference between the two extreme genotypes, the fittest genotype cannot invade the population. This result is in perfect agreement with numerically derived values in our model, e.g. a value of 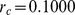 with the parameters of Figure 2. It is also in accord with the analytical result 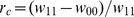 in the discrete time model [36], which translates to 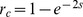 when Fisherian fitness is converted to the Malthusian fitness scale of our continuous time model. For small values of *s*, the difference between the critical recombination rates in the continuous vs. the discrete time model becomes very small.

For three loci, there are already 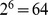 possible fitness landscapes, and in general no analytical result is available for these fitness landscapes. However, as we show in the Supplementary Online Material, for some special cases the dynamics can be understood in a simple way from the dynamics on the 2-locus landscapes (Figure S1 in Text S1).

### Deterministic Dynamics with Four Loci

We now consider the dynamics in the four-locus case. We define the following standard parameter set: 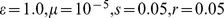. With this parameter set, there is no baseline epistasis and the fitness of the least fit genotype is 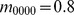. For a given parameter set, we screened all possible fitness landscapes with up to ten LFGs at intermediate genotypes. There are 14 intermediate genotypes and hence 
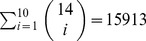
 possible configurations of LFGs. For each of these fitness topographies and each parameter combination, we measured the effect of recombination, *T*_fix_. Note that as in the two-locus case, high recombination rates may also prevent fixation of the fittest genotype on some fitness landscapes that are characterized by reciprocal sign epistasis. However, with the relatively low recombination rates that we assume here, the fittest genotype will always become fixed eventually and we therefore only focus on the time to fixation of that genotype rather than whether or not it becomes fixed.

**Figure 3:**
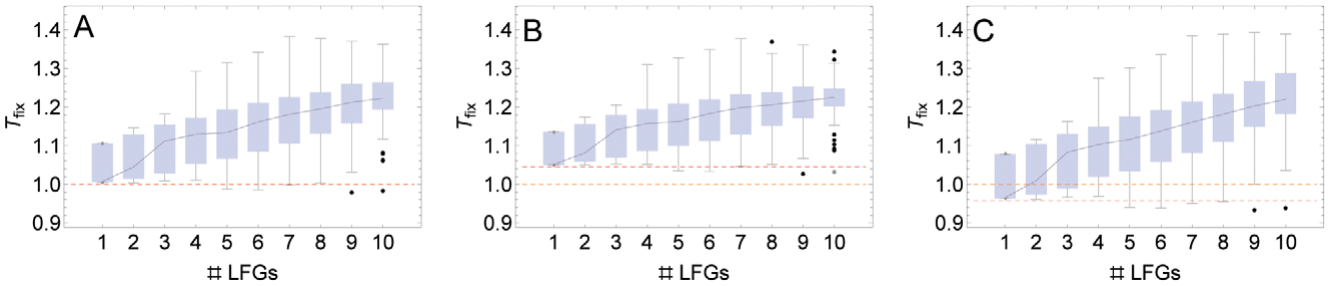
Effect of the number of LFGs in fitness landscapes on the relative rate of adaptation with recombination, *T_fix_*. A) no baseline epistasis (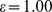), B) positive baseline epistasis (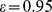), C) negative baseline epistasis (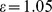). Each box shows the distribution of *T_fix_* across all fitness landscapes with the respective number of LFGs. The boxes give the interquartile range. Outliers are represented with the points in more than 1.5 times the interquartile range from the end of the boxes. The whiskers are extended to the farthest points from the end of the boxes that are not outliers. The black line connects the median of the boxes. The red dashed lines show *T_fix_* on the landscape with no LFG with the corresponding baseline epistasis. In the absence of baseline epistasis and LFGs in the fitness landscape, recombination has no effect on the rate of adaptation (*T_fix_* 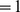, orange dashed lines). Parameters take the values 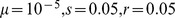.

We first investigate how the number of LFGs affects *T*_fix_ (Figure 3A). As expected, recombination has no effect on the rate of adaptation in the landscape without LFGs and without baseline epistasis (orange dashed lines). However, when LFG are introduced into the fitness landscape, recombination usually slows down the rate of adaptation. On average, this effect becomes stronger as the number of LFGs increases. This implies that the positive sign epistasis induced by LFGs has in general a similar qualitative effect as positive magnitude epistasis. However, there is substantial variance in *T*_fix_ across fitness landscapes with the same number of LFGs, indicating that the position of LFGs is crucial for the effect of recombination. There are even some fitness landscapes with a high number of LFGs where recombination has an accelerating effect. This clearly demonstrates that the above heuristic that positive epistasis produces a decelerating effect of recombination is not strictly valid (see also below and the Discussion).

When there is positive (negative) baseline epistasis, recombination decelerates (accelerates) adaptation in the landscape without LFGs (red dashed lines in Figure 3B and C). Introduction of LFGs again produces a decelerating effect of recombination (Figure 3B and 3C). For the parameter values chosen (relatively weak baseline epistasis), the effect of even a few LFGs in the fitness landscape generally outweighs the effect of negative baseline epistasis, so that overall, recombination usually has a decelerating effect (*T*_fix_ 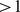). With a very high number of LFGs, *T*_fix_ becomes largely independent of the baseline epistasis.

**Figure 4:**
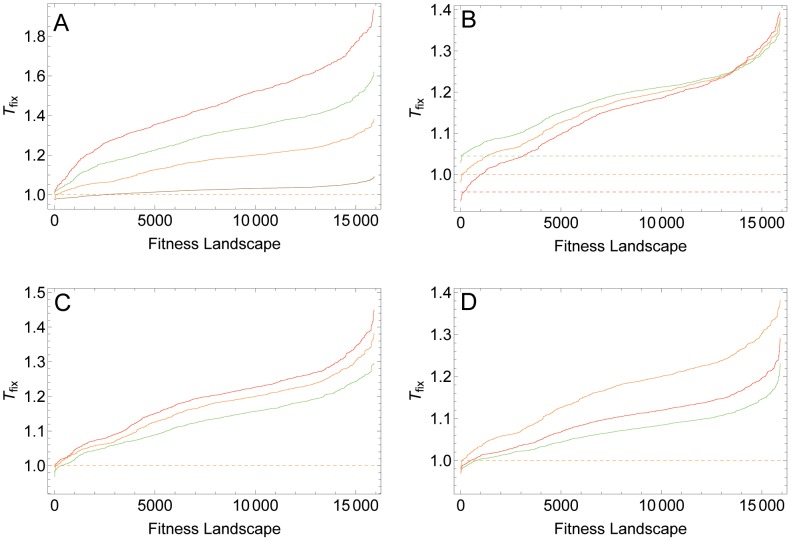
Effect of different parameters on *T*_fix_ on all possible four-locus fitness topographies with up to 10 LFGs. In all plots, the standard parameter set was used and one parameter was varied. Solid lines shows independently ranked *T*_fix_ values for all fitness topographies. For comparison, the dashed lines show *T*_fix_ in the corresponding fitness landscape with no LFG. A) Effect of recombination rate. Red, green, orange and brown curves correspond to *r* values of 0.1, 0.075, 0.05 and 0.01, respectively. B) Effect of baseline epistasis. Green, orange and red curves correspond to 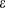 values of 0.95, 1.0 and 1.05, respectively. C) Effect of mutation rate. Red, orange and green curves correspond to 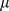 values of 10^−6^, 10^−5^ and 10^−4^, respectively. D) Effect of selection coefficient. Orange, red and green curves correspond to 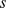 values of 0.050, 0.075 and 0.1, respectively. Note the different scales of the y-axes in plots A to D.

We next explored how the different parameters affect *T*_fix_. To this end, we again used our standard parameter set and varied one parameter while keeping the others constant. In most of our fitness landscapes, recombination decelerates adaptation and this effect becomes more pronounced with increasing recombination rate (Figure 4A). However, for very few fitness landscapes, recombination can also accelerate fixation of the fittest genotypes, and the number of fitness landscapes for which this holds increases with decreasing recombination rate. As expected, negative and positive baseline epistasis produces an accelerating and decelerating effect of recombination, respectively (Figure 4B). With higher mutation rates, the decelerating effect of recombination is reduced on most fitness landscapes, but there are also some landscapes where recombination has an accelerating effect with high mutation rates (Figure 4C). Recombination also has a weaker decelerating effect with higher baseline selection coefficients (Figure 4D).

**Figure 5:**
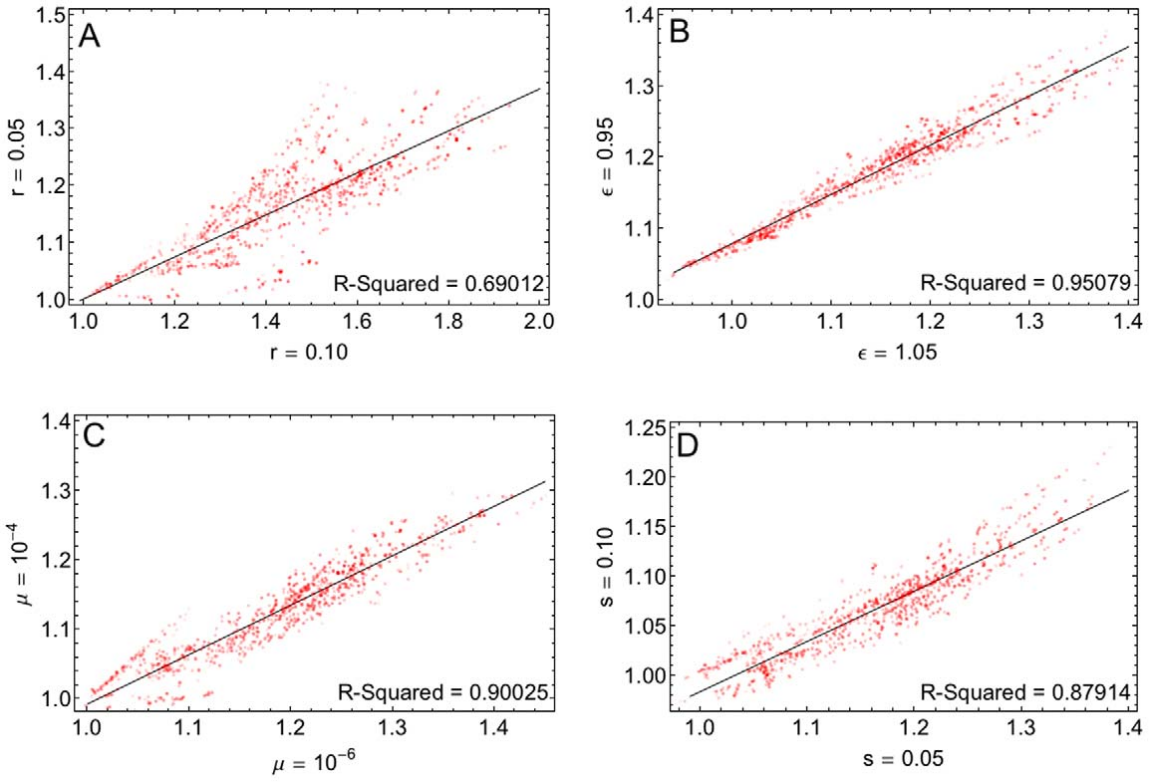
Robustness of the relative rate of adaptation *T_fix_* with regard to the parameters of the model: A) recombination rate, B) baseline epistasis, C) mutation rate and D) selection coefficient. Each point in the above plots represents one fitness topography and its position is given by *T_fix_* with two different parameter values. Other parameters take the same values as in Figure 4.

We can also ask to what extent the effect of recombination is a property of a specific fitness topography or an effect of other parameter values. In Figure 5, we plot corresponding *T*_fix_ values for two different parameters against each other. These plots indicate that the effect of recombination on the rate of adaptation is fairly robust with respect to the baseline selection coefficient, the mutation rate and the baseline epistasis parameter. However, we see that the effect of recombination rate can vary substantially for individual fitness topographies, and this variation is even more substantial in comparisons between more different recombination rates (e.g., we measured R-Squared 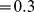 in the comparison between recombination rates 0.01 and 0.1). In the Supplementary Online Material (Figure S2 in Text S1), we further explore this observation and demonstrate that the recombination rate has a non-monotonic effect on the rate of adaptation [see also 32].

**Figure 6:**
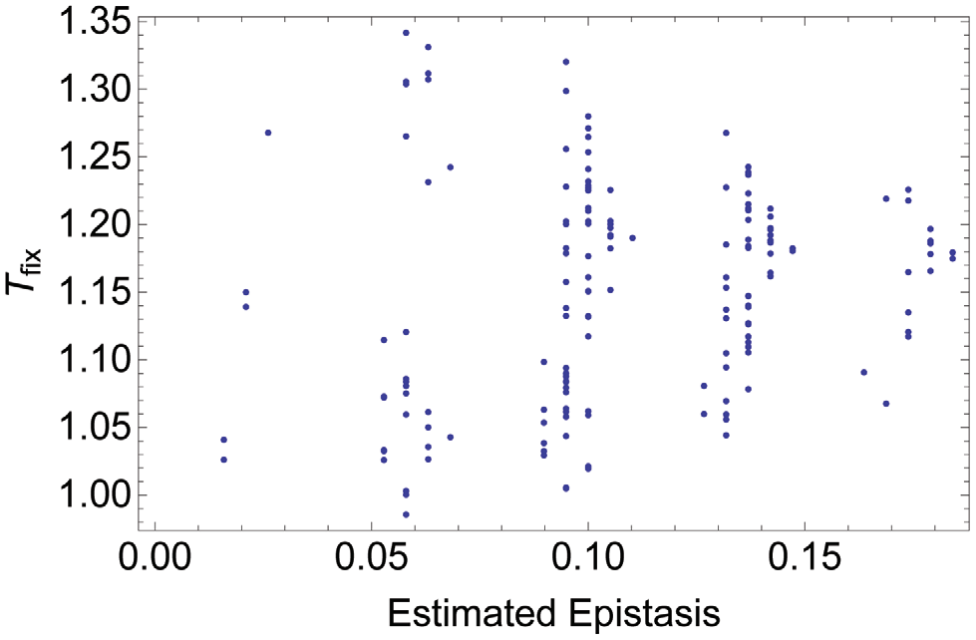
Scatter plot of estimated physiological epistasis against *T_fix_* for all fitness landscapes with 6 LFGs. Each point corresponds to one landscape. Parameters take values 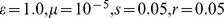. See main text for a description of how we measured physiological epistasis on these fitness landscapes.

Our results indicate that LFGs in the fitness landscape have an effect similar to positive magnitude epistasis in that recombination slows down adaptation. We therefore ascertained whether measured epistasis on our fitness landscapes is a predictor for the effect of recombination. To this end, we regressed fitness against the number of deleterious mutations 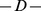 according to 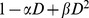, where 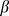 is an estimate for the (physiological) epistasis of the fitness landscape [7], [38]. Figure 6 plots the estimated epistasis values for all possible fitness landscapes with six LFGs against *T*_fix_. As anticipated, all landscapes are characterized by positive epistasis. However, there is no correlation between this measure of epistasis and the effect of recombination on adaptation, *T*_fix_. As an example, Figure S3 in Text S1 shows three landscapes with the same estimated epistasis value, but in which recombination has the most accelerating effect, no effect and the most decelerating effect compared to other fitness landscapes with six LFGs. This demonstrates the limitation of predictors based on measuring one-dimensional epistasis to predict the recombination effect on adaptation rate on complex fitness landscapes [see also 38].

### Stochastic Simulations

Due to computational limitations, an exhaustive study on all possible landscapes analogous to the deterministic part was not possible. Therefore, we randomly sampled 50 fitness topographies with 3, 5 and 7 LFGs and determined the fixation time for all of these topographies. We used the same standard parameter set as in the deterministic model. We focused on the region of the parameter space where 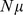 takes intermediate values, because this is where recombination is expected to have pronounced effects through finite population size (see Discussion).

**Figure 7:**
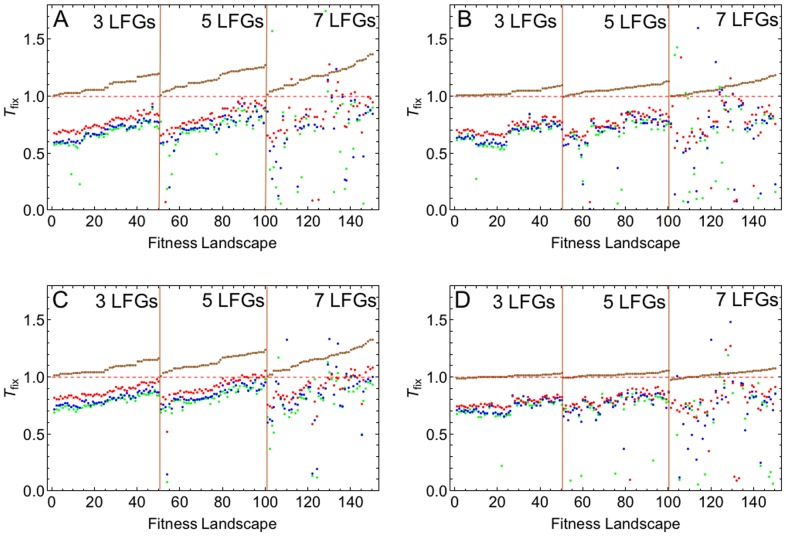
Effect of recombination on the rate of adaptation in finite populations. We screened a total of 150 randomly sampled fitness topographies with 3, 5 and 7 LFGs. *T_fix_* was determined for three different population sizes: 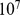 (red), 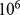 (blue) and 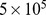 (green). All *T_fix_* values are sorted according to their recombination effect in the deterministic model (brown). Parameters take standard values (see also Figures 4 and 5), and in plots B to D we varies one of the parameters: A) Standard parameter set, B) 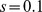, C) 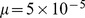 and D) 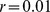.

Figure 7 shows the effect of recombination on the rate of adaptation in finite populations with our sample of fitness landscapes. It can be seen that in contrast to the deterministic case, recombination has predominantly an accelerating effect. Thus, even with relatively large population sizes, the accelerating Fisher-Muller effect due to finite population size outweighs the decelerating effect of recombination induced by epistasis. The accelerating effect of recombination becomes stronger with decreasing population size. (However, note that in line with previous studies [18], below a certain threshold of 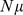, the Fisher-Muller effect disappears; see Figures S5 and S6 in Text S1).

It is also evident that at least for low numbers of LFGs in the fitness topographies (3 or 5), the ranking of *T_fix_* across fitness landscapes is the same with finite as with infinite populations, indicating that the stochastic and the deterministic effects are largely independent. When the fitness landscapes have many (7) LFGs, the ranking of *T_fix_* is no longer preserved. This is due to the fact that the variance in fixation times across replicates increases substantially (see Figure S7 in Text S1) because here, the time to fixation of the fittest genotype is dominated by the time that the populations spends in a monomorphic state on local fitness peaks (Results not shown). A higher mutation rate reduces this time and thereby restores the ordering of *T_fix_* values to some extent (Figure 7C).

Comparing the different panels in Figure 7, it can be seen that the baseline selection coefficient has only a minor effect on *T_fix_* (compare Figures 7A and B). Increasing the mutation rate has a similar effect as increasing population size in that it reduces the accelerating effect of recombination (compare Figures 7A and C). This is in line with previous results on the Fisher-Muller effect that stress the importance of 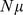 as compared to population size *per se* [18], [see also 48]]. Finally, with a low number of LFGs, decreasing the recombination rate weakens the Fisher-Muller effect (Figure 7D). As in the deterministic model, the effect of recombination rate here is also non-monotonic, i.e., there is an intermediate recombination rate at which adaptation is strongly accelerated (see also Figure S8 in Text S1). However, note that this effect, which was described in previous studies [37], [49], is distinct from the non-monotonic recombination effect observed in the deterministic setting.

## Discussion

We studied the effect of recombination on the tempo of adaptation. We focused on adaptation on adaptive fitness landscapes with limited peak accessibility, i.e., fitness landscapes with an underlying monotonic gradient of fitness values towards a single global fitness peak but where some genotypes have a very low fitness (see Figure 1B). Our approach was to numerically screen a large number of fitness landscapes in order to obtain a general view of the effect of recombination. We considered both a deterministic model (where linkage disequilibrium is solely generated through the epistatic effects implicit in the fitness landscape), and a stochastic model (where linkage disequilibrium is also generated through random mutation and genetic drift). We will discuss the results for both of these models in turn.

In the absence of random effects, recombination slows down adaptation on most fitness landscapes. This finding is consistent with analytical results for two-locus fitness landscapes exhibiting a fitness valley [4], [35]–[37], and also with a previous theoretical study on experimentally derived complex fitness landscapes [32]. Our results show that the higher the number of LFGs in the fitness landscape, the larger the decelerating effect of recombination becomes. This can in part be explained by the fact that a higher number of LFGs will generally produce a higher number of local fitness peaks. In a population occupying such a local fitness peak, recombination generally has a deleterious effect because it breaks down escape double mutant genotypes to genotypes occupying the fitness valley surrounding the local peak (for an example, see the fitness landscape in Figure S2A in Text S1). More generally, when there are many LFGs in the fitness landscape, the product of a recombination event between two genotypes is likely to be an LFG on the fitness landscape. Nevertheless, recombination can also have an accelerating effect (at least temporarily) during the course of adaptation whenever several viable genotypes coexist in the population among which recombination can produce a genotype of higher fitness (see Figure S2D in Text S1). The net effect of recombination will depend strongly not only on the fitness landscape, but also on the recombination rate: whereas modest recombination rates can sometimes accelerate adaptation, high recombination rates are usually detrimental. This non-monotonic influence of recombination rate was also previously reported [32], [49].

Unfortunately, it is very difficult to predict the impact of recombination on our as well as on other complex fitness landscapes from simple statistics derived from the landscape [38]. One statistic that has been frequently used as a predictor is the ‘physiological epistasis’, i.e., the curvature of the fitness effects with increasing number of deleterious or beneficial mutations from a reference sequence [7], [10], [50], [51]. We have also calculated this statistic for our fitness landscapes, but found that it has no predictive power with respect to the impact of recombination on the rate of adaptation. This is in line with a similar result on NK fitness landscapes [38]. Moreover, even on simpler multilocus fitness landscapes where only main effects and pairwise epistatic effects are considered, the physiological epistasis is a poor predictor when epistatic effects vary in strength and direction across loci ([52], see also [7]). Although we have not evaluated other predictors, we expect that no single statistic derived from the fitness landscapes in question exists that accurately predicts the effect of recombination.

The situation becomes more complicated when finite populations are considered. With stochastic mutation and random genetic drift, clonal interference between beneficial mutations at different loci can ensue, so that recombination can accelerate adaptation (the Fisher-Muller effect, which can be considered a special case of the Hill-Robertson effect [22]). In our model, the Fisher-Muller effect is sufficiently strong to outweigh the decelerating epistatic effects that arise from the structure of the fitness landscapes. Even with very large population sizes, recombination generally accelerates the adaptive process. This result is in accord with earlier works showing that recombination speeds up adaptation in bacterial populations [53]–[55]. Furthermore, the Hill-Robertson effect was shown to be strong enough that recombination can be selected for even in the presence of epistatic interactions between deleterious mutations when many loci are considered [56]. We also observed that the decelerating and accelerating impact of epistasis and stochastic effects are largely independent, as indicated by a roughly constant difference in the fixation times at different population sizes across all of our fitness topographies.

We have focused on a particular regime of the parameter space where selection is relatively strong and the number of mutations that arise in the population (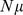) takes intermediate values (the strong selection strong mutation, or SSSM regime). This is the regime where clonal interference and hence recombination is important [57]. By contrast, when 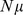 is small and selection is sufficiently strong (strong selection weak mutation, or SSWM regime), adaptation will proceed in sequential fixation of increasingly fit genotypes. In this case, there will not be any polymorphism at more than one locus simultaneously and therefore recombination has no effect [17], [20], [57]. Our results show that the exact boundary between the SSSM and the SSWM regime depends on the fitness landscape: when there are many LFGs in the landscape, clonal interference becomes less important for given 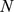 and μ. We expect that this is because with a higher number of LFGs, the number of possible beneficial mutations that are accessible by a given genotype becomes smaller. A final regime is the one where 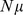 is very high. Here, all possible genotypes will be present in the population and thus, the stochastic model behaves like the deterministic model. (Note that in our model we consider recurrent mutations occurring at a finite number of loci. Therefore – unlike in models considering a potentially infinite number of beneficial mutations [17] – the accelerating effect of recombination is observed at intermediate population sizes; see [18] for a discussion of this effect.)

Our model was motivated by recent evolution experiments in bacteria (e.g., [27]) and therefore differs in two important aspects from traditional population genetics models investigating the consequences of recombination. First, our model is a continuous time model. This means that evolutionary parameters need to be interpreted in a slightly different way than in the standard Fisher-Wright model (e.g., Malthusian vs. Fisherian fitness), but otherwise we do not expect our continuous time assumption to affect our conclusions. Second and more importantly, we assume a bacterial mode of ‘piecewise’ recombination as seen in bacterial transformation where an allele in a recipient bacterium is replaced by a corresponding allele derived from a donor bacterium. This mode of recombination is equivalent to recombination through meiotic crossovers when there are only two loci, but is different with a larger number of loci. However, since we have not incorporated any other, more specific assumptions about bacterial recombination in our model (e.g., development of competence for transformation), we expect that our results are still very generic and should readily translate to eukaryotic or viral forms of recombination.

Only few studies are devoted to investigating the evolutionary effect of recombination on complex fitness landscapes. Here, we observed that including more features besides steepness and curvature in the structure of fitness landscapes results in rich dynamics and complex effects of recombination on the evolutionary process. More work is necessary to elucidate what properties of fitness landscapes are decisive for the impact of recombination and to quantify those properties in empirical fitness landscapes.

## Supporting Information

Text S1 Supplemental figures mentioned in the text.
(PDF)
