# Supplementary material for: The Effect of Bacterial Recombination on Adaptation on Fitness Landscapes with Limited Peak Accessibility
Source: PLoS Comput Biol. 2012 Oct 25;8(10):e1002735. doi: 10.1371/journal.pcbi.1002735 (PMC3487459; doi:10.1371/journal.pcbi.1002735)
Supplement: Text S1 — Supplemental figures mentioned in the text. (PDF) [file pcbi.1002735.s001.pdf]

Supplementary Online Material

Contents:

1. Reduction from three to two loci in the deterministic model 1
2. Examples for deterministic dynamics with four loci 2
3. Additional results in the stochastic model 5
4. The relation between population mean fitness increase and T_{fix} 9

1. Reduction from three to two loci in the deterministic model

In general it is very difficult to obtain analytical results for the dynamics of our model when there are more than two loci. However, for some fitness topographies the dynamical system behaves similarly to a system with a lower number of loci. As an example, we will consider here the 3-locus fitness topography shown in Figure S1. Here, all intermediate genotypes lacking the beneficial mutation at the third locus are LFGs on the fitness landscape. As a consequence, with high recombination rates adaptation proceeds in the initial fixation of the "001" genotype followed by the dynamics on two-locus fitness landscape with no LFG. To understand this process in mathematical terms, we analyze the pairwise LDs, defined as

$$D_{1,2} = (x_{000} + x_{001})(x_{110} + x_{111}) - (x_{010} + x_{011})(x_{100} + x_{101}), \quad (1a)$$

$$D_{2,3} = (x_{000} + x_{100})(x_{011} + x_{111}) - (x_{001} + x_{101})(x_{010} + x_{110}), \quad (1b)$$

$$D_{1,3} = (x_{000} + x_{010})(x_{101} + x_{111}) - (x_{001} + x_{011})(x_{100} + x_{110}). \quad (1c)$$

We assume a high rate of recombination ($r \to \infty$) so that all pairwise LDs are completely purged ($D_{1,2} = D_{2,3} = D_{1,3} = 0$). Furthermore, we assume that the frequencies of LFGs are so small that they can be neglected ($x_{100} = x_{010} = x_{110} = 0$). Under these assumptions, equations (1) read

$$D_{1,2} = (x_{000} + x_{001})x_{111} - x_{011}x_{101} = 0, \quad (2a)$$

$$D_{2,3} = x_{000}(x_{011} + x_{111}) = 0, \quad (2b)$$

$$D_{1,3} = x_{000}(x_{101} + x_{111}) = 0. \quad (2c)$$

Since the population starts from the state where $x_{000} > 0$, equations (2b) and (2c) imply that $x_{011} + x_{111} = 0$ and $x_{101} + x_{111} = 0$ must hold, which means that $x_{011} = x_{111} = x_{101} = 0$. Since all frequencies should add up to one, we have $x_{000} + x_{001} = 1 - x_{101} - x_{011} - x_{111}$ and therefore $x_{000} + x_{001} = 1$. Thus, in the first phase only the two genotypes "000" and "001" exist. However, due to selection for the "001" genotype, the frequency of the "000" genotype decreases to zero whereas frequency of the "100" genotype increases towards 1. Under this condition, equation (2a) changes to

$$D_{1,2} = x_{001}x_{111} - x_{011}x_{101} = 0, \quad (3)$$

which is the LD for the two-locus case. Figure S1 illustrates that the dynamics follow indeed the two-step process outlined above when the recombination rate is high ($r = 1.00$); for lower recombination rates, the dynamics are more complex.

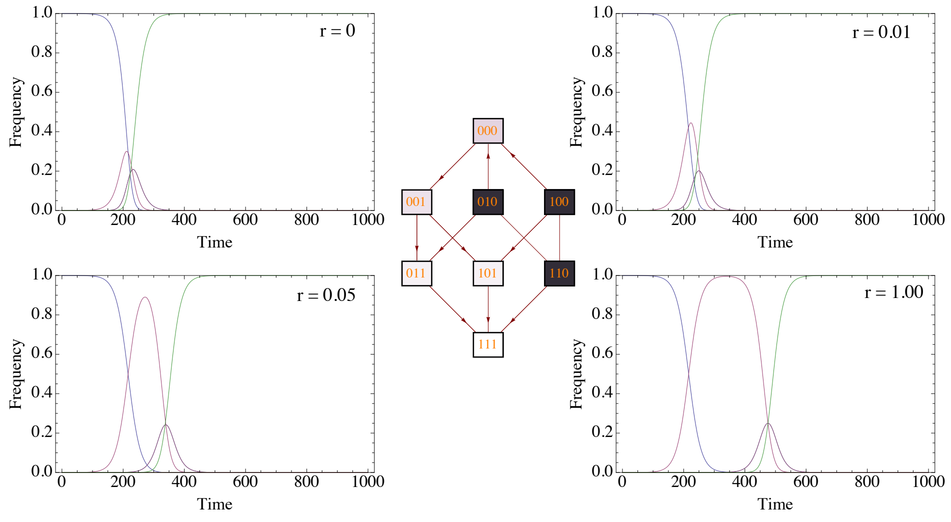

Figure S1. Reduction of population dynamics to the sequence of fixation of the non-LFG at the first layer and two-locus dynamics as recombination rate increases for the landscape shown above. Arrows in the landscape show point mutation steps directed toward fitter genotypes. The parameter set is $\epsilon = 1.0, \mu = 10^{-6}, s = 0.05$.

2. Examples for deterministic dynamics with four loci

Figure S2 illustrates the dynamics on one particular fitness landscape in which recombination can accelerate the rate of adaptation. This fitness landscape is characterized by a local fitness peak and by the lack of a mutational trajectory with monotonic fitness increases from the least fit to the fittest genotype (Figure S2A). Adaptation can still proceed through rare multiple consecutive mutations (provided that mutation rate is high enough) which enables the population to pass through the LFGs. Arrows in Figure S2B show the frequency of the "1000" genotype (occupying the local fitness peak) over time in the recombining and non-recombining population. During adaptation, recombination breaks up rare newly formed LFGs ("1100", "1010", "1001", "0100", "0010" and "0001"), by shuffling them with the more frequent genotypes "1000", "0000" and converting them to either the more frequent "0000" and "1000" genotypes or to other LFGs. In doing so, recombination makes the escape from the local peak genotype "1000" more difficult, as shown in Figure S2C. Nevertheless, the net effect of recombination on adaptation rate is not monotonic (Figure S2D), showing that depending on the standing polymorphism in the population, recombination can both accelerate and decelerate adaptation at different times during the evolutionary process.

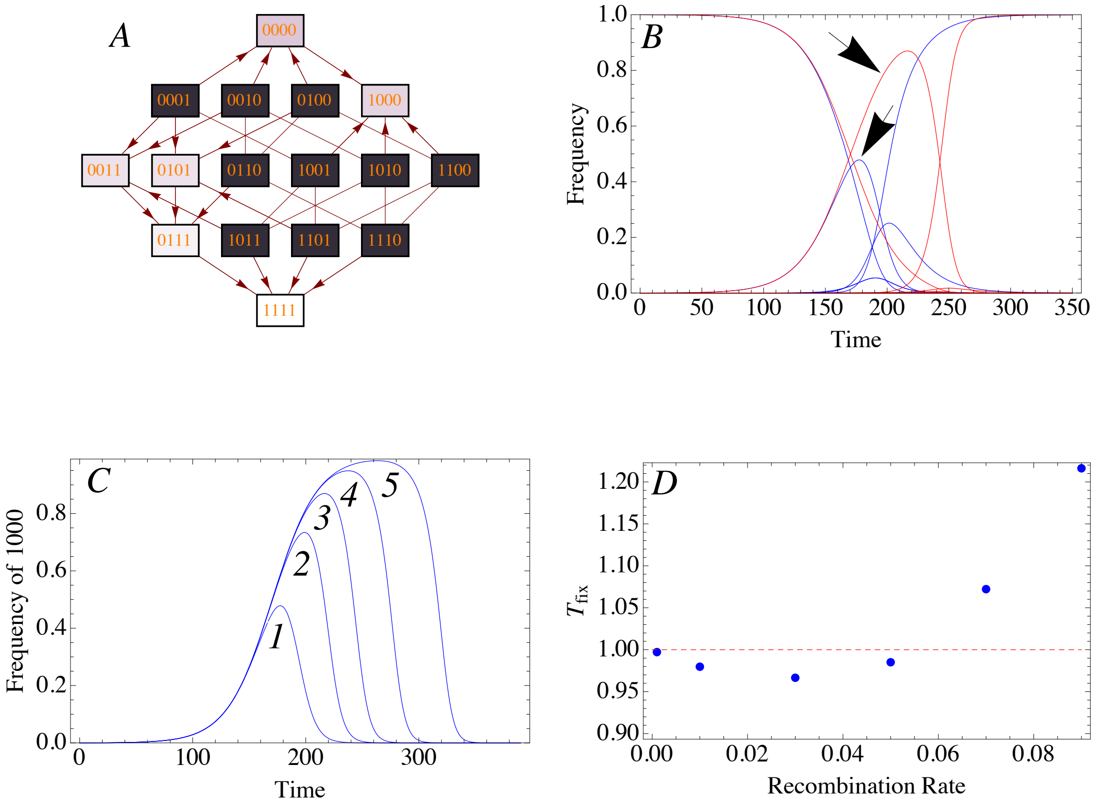

Figure S2. A) A fitness landscape with ten LFGs. Arrows show point mutation steps directed toward fitter genotypes. B) The dynamics of recombining (red) and non recombining (blue) populations with the parameter set $\epsilon = 1.0, \mu = 10^{-5}, s = 0.05, r = 0.05$. Each curve shows the frequency of one of the intermediate genotypes over time until the fixation of the fittest genotype “1111”. C) The dynamics of the frequency of the genotye “1000” (occupying the local fitness peak) with increasing recombination rates ($r_1 = 0, r_2 = 0.03\,, r_3 = 0.05\,, r_4 = 0.07\,, r_5 = 0.09$). D) Non-monotonic recombination effect on the time to fixation of the fittest genotype.

As shown in the main text, the physiological epistasis of a given fitness landscape is a poor predictor for the effect of recombination on the rate of adaptation. Figure S3 shows three examples of 4-locus fitness landscapes with the same number of LFGs in each layer (i.e., the same physiological epistasis), but with strongly divergent impact of recombination. This illustrates that the position of the LFGs relative to each other is important for whether recombination accelerates or decelerates the adaptive process.

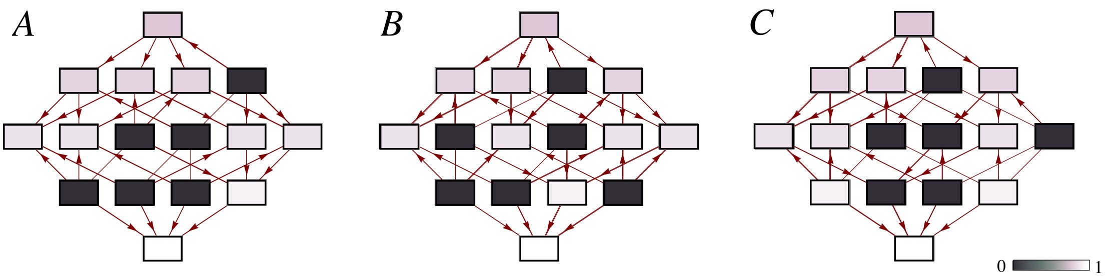

Figure S3. Fitness landscapes with the same physiological epistasis but with different effect of recombination in the deterministic model: recombination has (A) the most accelerating, (B) almost no and (C) the most decelerating effect compared to other fitness topographies with six LFGs. Arrows show point mutation steps directed toward fitter genotypes. Parameters take the values $\epsilon = 1.0$, $\mu = 10^{-5}$, $s = 0.05$, $r = 0.05$.

3. Additional results for the stochastic model

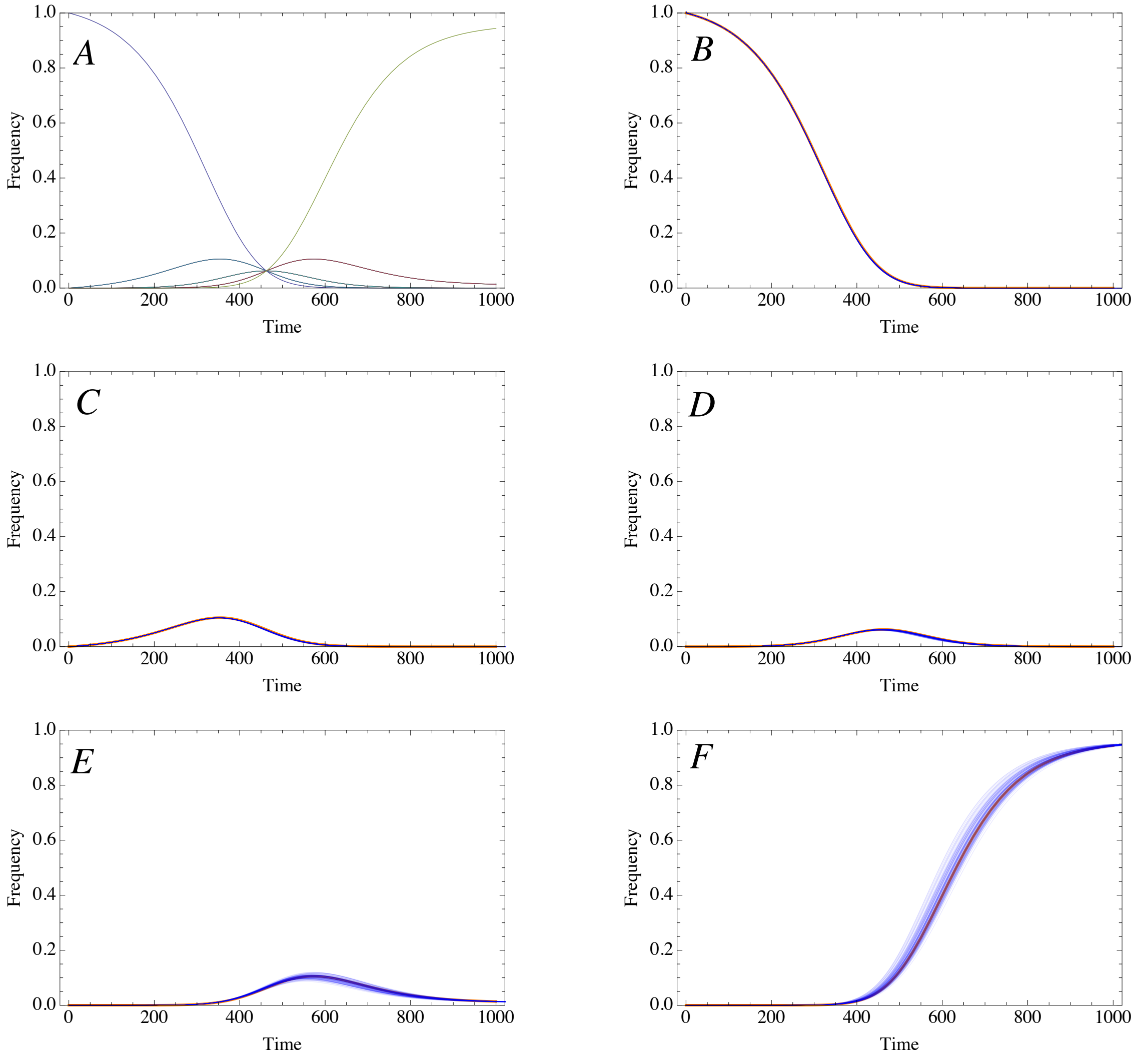

Figure S4. Validation of the stochastic model for large population size and weak selection. Panel A shows the dynamics of population on a four-locus landscape with no LFG in the deterministic model. Each line corresponds to a genotype. Panels B-F show the agreement between the stochastic model (blue curves, 100 simulations) and the deterministic model (red curves) for the five layers. Note that genotypes with the same number of beneficial mutations have the same dynamics for the landscape with no LFG. Parameters: $\epsilon = 1.0, \mu = 10^{-4}, N = 10^9, s = 0.01$.

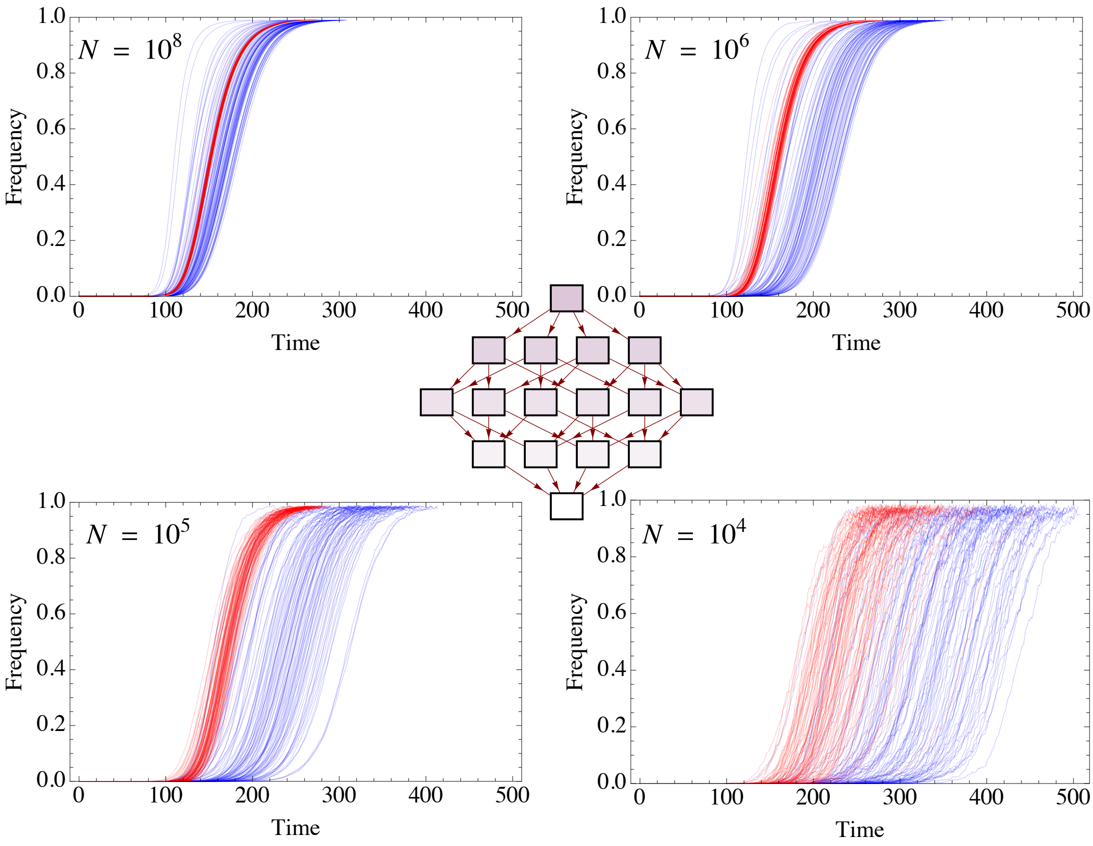

Figure S5. The Fisher-Muller effect on a fitness landscape with no LFG (shown in the middle). The red and blue curves correspond to the frequency of the fittest genotype in 100 simulations with and without recombination, respectively. Arrows in the landscape show point mutation steps directed toward fitter genotypes. The Fisher-Muller effect is most pronounced for intermediate values of $N\mu$. Parameters: $\epsilon = 1.0, \mu = 10^{-4}, r = 0.05\,, s = 0.05$.

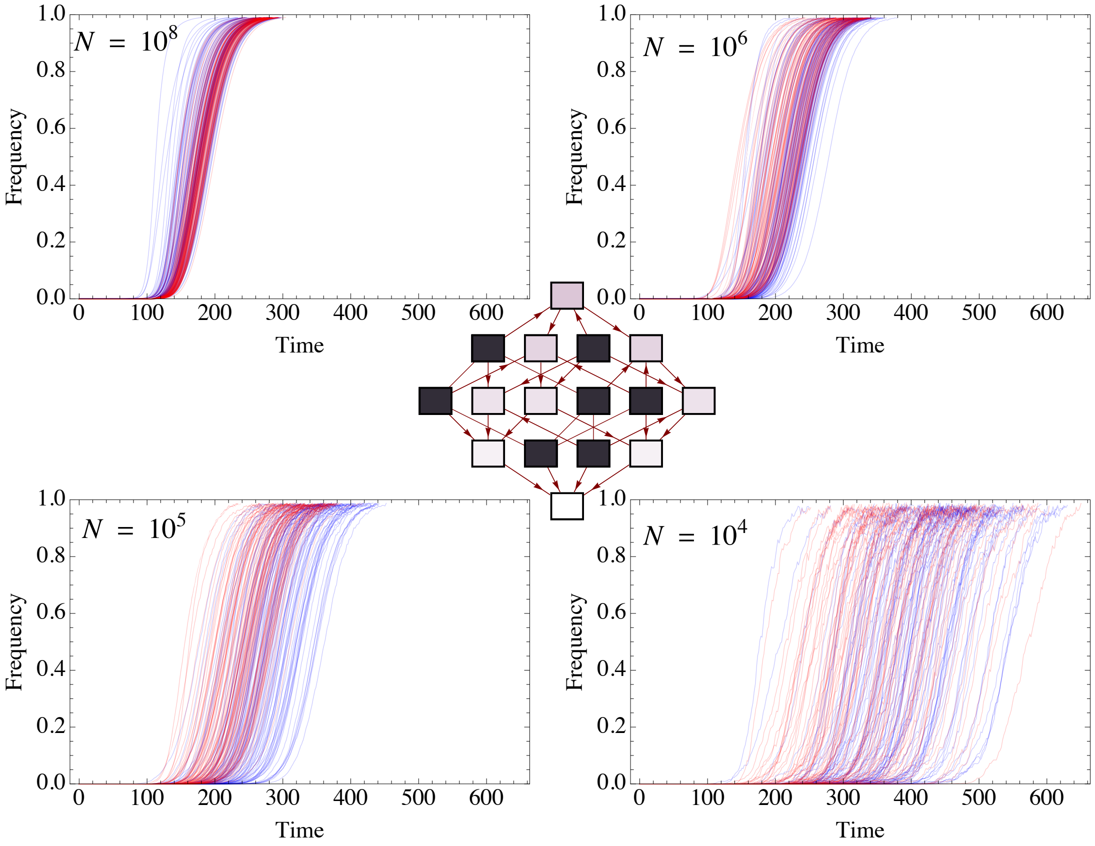

Figure S6. The Fisher-Muller effect on fitness landscape with 7 LFGs (shown in the middle). The red and blue curves correspond to the frequency of the fittest genotype in 100 simulations with and without recombination, respectively. Arrows in the landscape show point mutation steps directed toward fitter genotypes. Like on the fitness landscape with no LFG which is shown in Figure S5, the Fisher-Muller effect is most pronounced at intermediate values of $N\mu$. Parameters: $\epsilon = 1.0, \mu = 10^{-4}, r = 0.05, s = 0.05$.

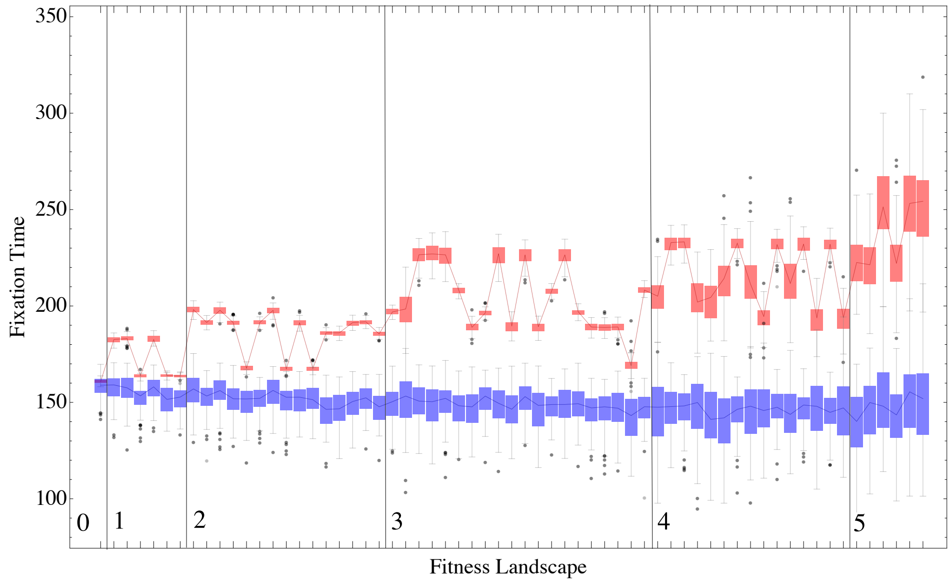

Figure S7. Variation in fixation times in on all topographies of fitness landscapes with LFGs with three loci. Red and blue boxes correspond to fixation times of 100 simulations for recombining and non-recombining populations, respectively. The boxes give the interquartile range. Outliers are represented with the points in more than 1.5 times the interquartile range from the end of the boxes. The whiskers are extended to the farthest points from the end of the boxes that are not outliers. The black line connects the median of the boxes. Fitness landscapes are ordered in blocks according to the number of LFGs they exhibit (numbers from 0 to 5). Note that because of the high mutation rate, the Fisher-Muller effect is not pronounced. At higher number of LFGs, the variance in fixation times generally increases for both recombining and non-recombining populations. Parameters: $\epsilon = 1.0, \mu = 10^{-3}, N = 10^{6}, s = 0.05, r = 0.10$.

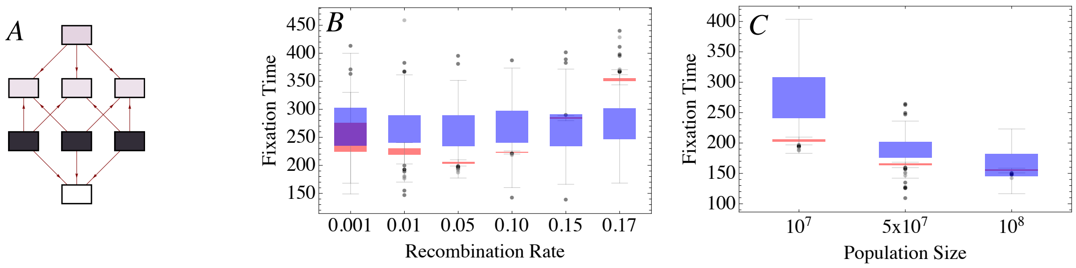

Figure S8. Effect of recombination in the stochastic model on a landscape with a valley at the penultimate layer. A) Landscape topology. Arrows show point mutation steps directed toward fitter genotypes. B) Nonmonotonic effect of the recombination rate on the times to fixation in the recombining population. The blue and red boxes show the fixation times in the non-recombining and recombining populations of 100 simulations, respectively. With $r > 0.17$, recombination prevents fixation of the fittest genotype. Other parameters take the values $\epsilon = 1.0, \mu = 10^{-4}, N = 10^{7}, s = 0.05$. Note this non-monotonic behavior is distinct from the one observed in the deterministic model (see e.g. Figure S2); the recombination effect for this fitness landscape in the

deterministic model is monotonically increasing. C) The effect of population size. At large population size Fisher-Muller effect is reduced. The parameter set is $\epsilon = 1.0, \mu = 10^{-4}, s = 0.05, r = 0.05$.

4. The relation between population mean fitness increase and T_{fix}

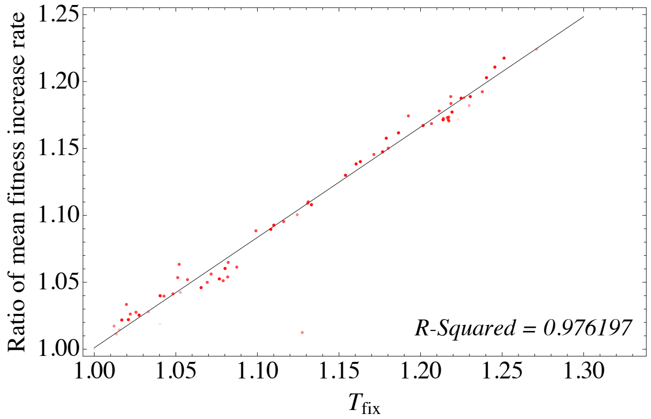

Figure S9 The ratio of the time, at which the population mean fitness exceeds the value of 0.99, in the recombining population to that in the non-recombining population plotted against the T_{fix}s for all landscapes with four LFGs. Each point in the plot represents one fitness landscape topography. The parameter set is: $\epsilon = 1.0, \mu = 10^{-5}, s = 0.05, r = 0.05$.
